# Why did Amelita Galli-Curci lose her voice?

**DOI:** 10.20945/2359-3997000000162

**Published:** 2019-08-14

**Authors:** Oscar D. Bruno

**Affiliations:** 1 Universidad de Buenos Aires University of Buenos Aires Buenos Aires Argentina University of Buenos Aires. President of the Foundation of Endocrinology (FUNDAENDO), Buenos Aires, Argentina

The main reason that led me to write this paper was the presentation of a poster at the 12^th^ International Thyroid Conference in Kyoto, Japan (2000), about “Complications of Thyroidectomy in Patients with Radiation-Induced Thyroid Neoplasms”. This poster was illustrated with a picture of the well-known opera singer Amelita Galli-Curci (1) (Figure 1). At that time, I became aware of what was called among American neck surgeons “the nerve of Galli-Curci”. But, what was the story behind it?

**Figure 1:**
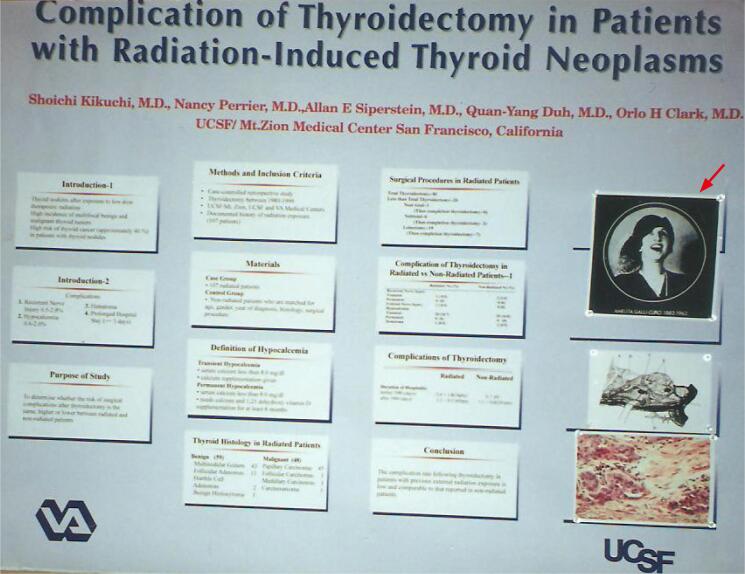
Poster presented by Kikuchi and cols. at the 12th International Thyroid Conference 2000 in Kyoto; the red arrow pointed at a portrait of AG-C, by then a symbol of complications of thyroid surgery.

## PERSONAL BACKGROUND

**Figure 2:**
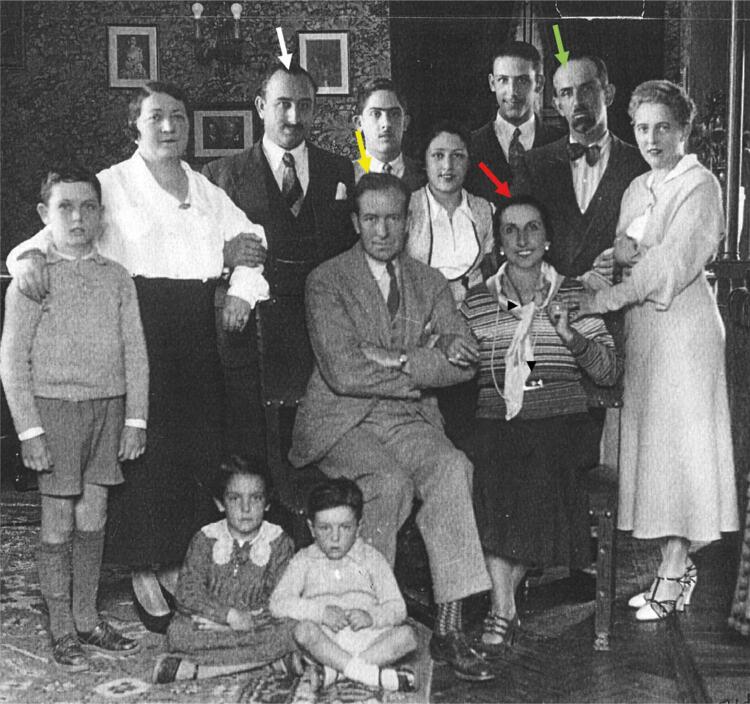
Family picture taken in Rosario in one of the visits of AG-C to Argentina. Red arrow, AG-C; yellow arrow, husband Homer Samuels; white arrow, brother Giuseppe; green arrow, brother Enrico.

Amelita GALLI BELLISOMI (born in Milan, Italy, in the year of 1882 and died in 1963, in La Jolla, California, USA) was a prestigious coloratura soprano from the first half of the 20^th^ century, well-known over the world. In her youth, she wished to become a piano soloist, but after an audition with a family friend, Pietro Mascagni (composer of Cavalleria Rusticana, L’amico Fritz, Le maschere, among others), he convinced her to pursue a career of opera singer. She made her operatic debut at Trani, as Gilda in Giuseppe Verdi’s Rigoletto*,* and from that time onwards, she became very popular and widely acclaimed by critics and public in Italy. She married an aristocratic man, Marquis Luigi Curci in 1908, when she adopted the hyphenation Galli-Curci (AG-C) along all her singing life, despite their divorce in 1920. She toured Europe, Russia and South America. In Buenos Aires, she performed twice with Enrico Caruso at The Colon Theatre, in 1915 (2). She travelled to Argentina several times due to professional engagements, but also probably because a significant part of her family lived there. We have to underline that, after an economic crash, her father migrated to Argentina. Her two brothers, Enrico and Giuseppe, also artistically well-endowed, used to live in the city of Rosario (Argentina) (Figure 2).

One of the stories involving Amelita (not verified) tells that in her last trip to Buenos Aires she decided to go to North America, because as World War I had broken out, there would be a significant risk of an attack to the ship that would take her back to Europe.

**Figure 3:**
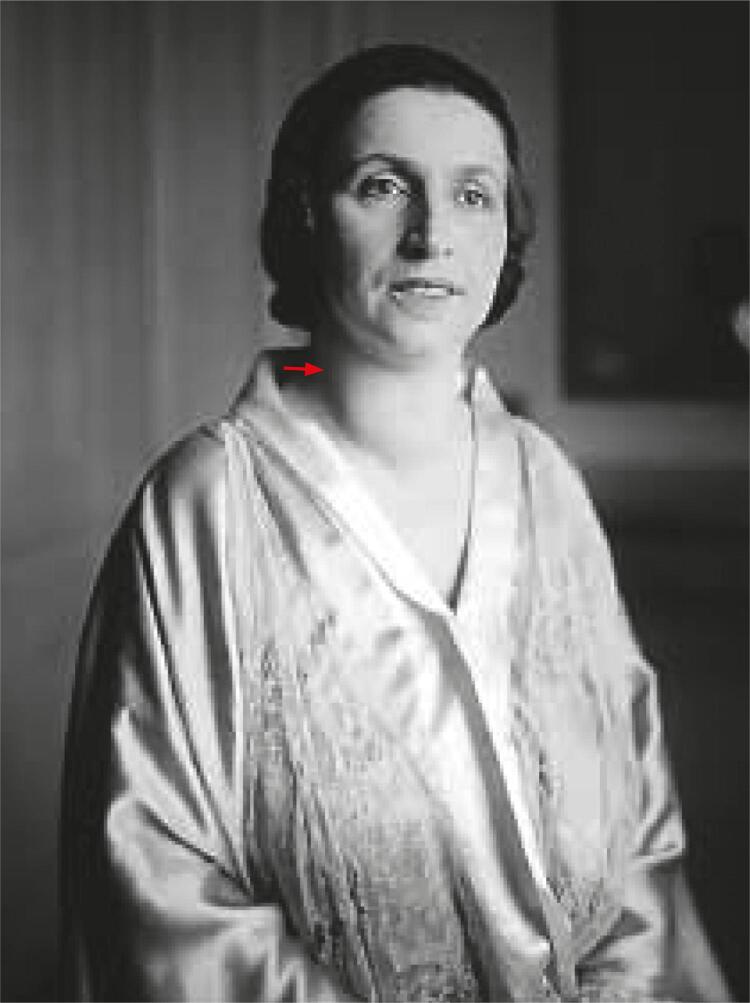
The red arrow points at Galli-Curci’s lump on the right side of her neck.

She sang for the first time at the Metropolitan Opera House (MET) in New York in 1916. Right after that she became engaged with the Opera Theatre of Chicago for several years, before becoming the main soprano at the MET of New York, from 1920 to 1930, when Amelita decided to give up opera singing and start giving recitals in different parts of the world (2). In 1935 she travelled with her second husband, Homer Samuels (her pianist partner), across India. In New Delhi she was affected by a worrisome dysphonia. Amelita had been suffering from a progressive asymmetric enlargement of the neck for fifteen years (Figure 3) and, therefore, she attributed her voice problems to this lump. At that time she heard that a prestigious American surgeon, Dr. Arnold H. Kegel, was performing epidemiologic studies about goiter in India and decided to consult him. She continued touring Asia accompanied by the surgeon, who observed how the goiter influenced her vocal performance. After obtaining an X-ray test in Tokyo, he determined that there was a reduction of around 50% in her tracheal caliber due to compression by the goiter, hence, he proposed a surgical treatment (2).

**Figure 4:**
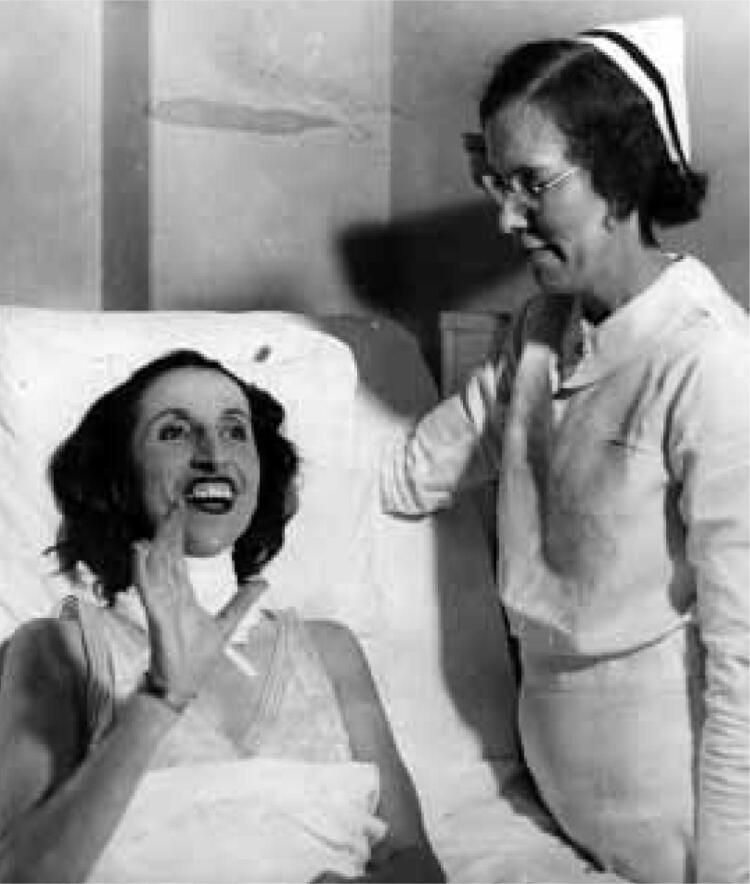
Galli-Curci in hospital after surgery (New York Public Library for the Performing Arts and Associated Press).

Back in the US (in Chicago), Amelita underwent surgery under local anesthesia, which allowed testing her vocal organs during the procedure. The “potato” (as she called it), which measured 11 x 5 cm and weighed 184 g was removed. The patient was happy (Figure 4) and declared that *“the result of the operation is just short of marvelous. My voice is like a young colt - I will have to restrain it”* (2). However, she also declared to the Time Magazine: “*The operation has pulled my voice way down and I would like to come back to opera as a lyric soprano instead of a coloratura”.* There is some controversy on whether Galli-Curci’s voice improved after surgery or, if on the contrary, she progressively lost his singing skills. On the one hand, there are some authors who advocate for the opposite (3). On the other, after her first post-surgery operatic performance in 1936, in Chicago, as Mimi in *La Bohème*, the critics unanimously commented on her lack of upper range, inability to sustain notes and notorious breathlessness: “*She had command neither of voice nor of breath*” wrote Eugene Stinson, of the “Daily News”. She gave some recitals during one more year and then she retired at the age of 54 (2).

## MEDICAL HISTORY (THE NERVE OF GALLI-CURCI: MYTH OR REALITY?)

Those who concluded that Galli-Curci was affected by thyroid surgery explained that the damage to the external branch of the superior laryngeal nerve which innervates the crico-thyroideal muscle, derived in her inability to sing and sustain high pitches; so, this nerve has become to be known since then as the “nerve of Galli-Curci”*.* Some other specialists did not agree with that interpretation. Instead, they attributed her vocal decline to the effect of aging as it happened to others sopranos who changed their vocal registries from coloratura to dramatic as happened with Joan Sutherland or Montserrat Caballé (3).

But there is another possible explanation which was briefly evoked by some authors (4): the possibility that Amelita remained hypothyroid after surgery. Thyroid hormone receptors are present in the larynx and hypothyroidism is well known to affect voice (5,6). A severe lack of thyroid hormones is manifested by a hoarse voice. Although there is no information about the extension of the thyroidectomy, it was probably significant, given the weight (184 g) of what was removed during surgery. It has been quoted that after the operation she had a suggestive 15 pounds increase in body weight (4). At that time, diagnosis of thyroid insufficiency was mainly based on clinical grounds and on the measurement of basal metabolic rate which allowed diagnosis of obvious myxedema states (severe hypothyroidism), but not of less evident deficiencies. Although we do not know how she was treated, there is a paucity of data in the literature on the effect of subtle deficiency on vocal abilities, mainly in professional singers who naturally might be at risk. A harsh voice amongst more severe signs of hypothyroidism has been correlated with the presence of antityroglobulin antibodies (ATG) (7), but we do not know if Amelita also had autoimmune thyroiditis, since ATG levels were not measured in 1935 and the thyroid pathology failed to reveal it. Moreover, adjustment of thyroid reposition through the measurement of serum protein bound iodine (PBI) was only introduced in the forties (8,9) and that of thyroid stimulating hormone in the sixties (10). Since 1960, thyrotropin releasing hormone (TRH) tests have allowed the detection of more slight deficiencies (8) and, nowadays, ultrasensitive immunoradiometric thyroid-stimulating hormone (TSH) assays using monoclonal antibodies allow an even more delicate control of thyroid therapy, having replaced TRH-TSH tests (11). This control seems to be optimized in some cases through the measurement of serum levels of free triiodothyronine (12).

There is no information about either her being treated with thyroid extracts or about how she was clinically and biochemically controlled. In this respect, it is worth remembering that it was Murray, in 1891, known as the father of endocrine-replacement therapy, who first described the cure for hypothyroidism with the administration of sheep thyroid extracts. In 1940 Means (13) wrote: “*The treatment of the usual type of myxedema is so simple that we need not devote much time to it. The objective should be to rid the patient of his symptoms and clinical signs with the smallest daily ration of thyroid by mouth that will accomplish this purpose. Usually this will be found to be in the neighborhood of one to one and a half grains of thyroid once daily. If such dosage produces any sort of untoward symptoms, angina or other, thyroid should be stopped for a few days and then resumed in smaller dosage”.* Nowadays we know that things are not that easy…Treatment for thyroid deficiency has continued to evolve during two thousand years, from the therapy of Chinese cretins with sheep thyroids in the 6^th^ century to the purified preparations of levothyroxine employed presently. A detailed historical review of the evolution of the hypothyroidism treatment can be found in two excellent publications (14,15). At the time Amelia underwent surgery and during the following years, the usual therapy consisted in the administration of USP grains of desiccated thyroid extracts. Control and adjustment of therapy was based on clinical grounds, such as body weight, edema, pulse rate, skin quality, sensitivity to cold and measurement of basal metabolic rate or Achilles tendon relaxation time.

Therefore, it is clear that in those years diagnosis, evaluation, thyroid hormone substitution and control of therapy of the hypothyroid status were rudimentary compared to our days and that a persistent moderate or subtle deficiency could have been the cause of the vocal decline of Amelita Galli-Curci.

In summary, we cannot attribute her loss of vocal performance either to the superior laryngeal nerve lesion or to aging, but rather to the possibility of hypothyroidism due to an imperfectly regulated thyroid replacement. This is only an imaginative hypothesis and, to conclude with a well-known Italian phrase as a tribute to Amelita Galli-Curci: “*si non è vero è ben trovato…”* (if it is not true, it is well invented…).
